# Culturable Yeast Diversity Associated with Industrial Cultures of the Microalga *Microchloropsis gaditana* and Their Ability to Produce Lipids and Biosurfactants

**DOI:** 10.3390/jof11030228

**Published:** 2025-03-17

**Authors:** Madalena Matos, Mónica A. Fernandes, Inês Costa, Natacha Coelho, Tamára F. Santos, Veronica Rossetto, João Varela, Isabel Sá-Correia

**Affiliations:** 1 iBB—Institute for Bioengineering and Biosciences, Instituto Superior Técnico, Universidade de Lisboa, Av. Rovisco Pais, 1, 1049-001 Lisbon, Portugal; madalena.matos@tecnico.ulisboa.pt (M.M.); monica.a.fernandes@tecnico.ulisboa.pt (M.A.F.); 2 Associate Laboratory i4HB—Institute for Health and Bioeconomy, Instituto Superior Técnico, Universidade de Lisboa, Av. Rovisco Pais, 1049-001 Lisbon, Portugal; 3 Necton S.A., Belamandil, 8700-152 Olhão, Portugal; ines.costa@necton.pt (I.C.); natacha.coelho@necton.pt (N.C.); 4 MED—Instituto Mediterrâneo para a Agricultura, Ambiente e Desenvolvimento, CHANGE—Global Change and Sustainability Institute, Faculdade de Ciências e Tecnologia, Campus de Gambelas, Universidade do Algarve, Ed. 8, 8005-139 Faro, Portugal; 5 Centre of Marine Sciences, Campus Gambelas, University of Algarve, 8005-139 Faro, Portugal; tfsantos@ualg.pt (T.F.S.); vrossetto@ualg.pt (V.R.); jvarela@ualg.pt (J.V.); 6 GreenCoLab—Associação Oceano Verde, Campus Gambelas, University of Algarve, 8005-139 Faro, Portugal; 7 Department of Bioengineering, Instituto Superior Técnico, Universidade de Lisboa, Av. Rovisco Pais, 1, 1049-001 Lisbon, Portugal

**Keywords:** yeasts, yeast isolation, microalgae cultivation, *Rhodotorula*, Basidiomycota, biotechnological applications

## Abstract

The marine oleaginous microalga *Microchloropsis gaditana* (formerly *Nannochloropsis gaditana*) exhibits a high capacity to thrive in a broad range of environmental conditions, being predominantly utilized as feed in aquaculture. This article reports the characterization of the culturable yeast population present during the scale-up process of *M. gaditana* cultivation at Necton S.A. facilities, from 5 L flasks until tubular photobioreactors. The 146 yeast isolates obtained, molecularly identified based on D1/D2 and ITS nucleotide sequences, belong to the species *Rhodotorula diobovata*, *R. mucilaginosa*, *R. taiwanensis*, *R. sphaerocarpa*, *Vishniacozyma carnescens*, *Moesziomyces aphidis*, and *Meyerozyma guilliermondii*. The yeast abundance was found to increase throughout upscaling stages. The yeast populations isolated from microalgal cultures and water samples share phylogenetically close isolates, indicating a possible common source. The impressive high percentage of red yeasts isolated (90%) is consistent with the recognized role of carotenoid pigments in yeast photoprotection. Sixty yeast isolates were tested for lipid (Nile Red staining) and biosurfactant (oil drop dispersion and emulsification index) production. Results revealed that these capacities are common features. Microbial lipids and biosurfactants have promising biotechnological applications. Moreover, biosurfactants can fulfill various physiological roles and provide advantages in natural environments contributing to the promising use of yeasts as probiotics in microalgae production.

## 1. Introduction

The demand for large-scale production of microalgae is increasing [1]. These photosynthetic unicellular or colonial microorganisms are the base of the entire aquatic food chain [1]. Microalgae are known producers of value-added bioproducts (e.g., polysaccharides, lipids, biosurfactants, pigments, vitamins, antioxidants) of interest for the cosmeceutical, nutraceutical, food and agricultural industries [1,2,3,4]. In particular, they are relevant for the development of healthy foods and food supplements, feeds, biofertilizers, biostimulants, or biopesticides [1,2,3,4]. Microalgal biomass generation is commonly achieved through open systems (open or raceway ponds) or closed systems (photobioreactors such as flat panels or tubular production systems) [1,5]. Regardless of the biomass production system used, biological contamination is one of the most critical problems associated with large-scale algal production, especially in open systems, which require significantly lower capital and operational costs [5,6]. The presence of biological contaminants can often cause a decrease in the production of biomass and products of interest, possibly leading to culture collapse and loss of commercial value [7,8]. However, recent studies indicate that, in natural habitats, the microalgal microbiome may play a key role in modulating algal populations [7]. For example, in industrial settings, the microalgal symbionts might promote algal growth, the synthesis of added value bioproducts, and offer advantages in downstream processing [7,9,10]. Nonetheless, there is a lack of comprehensive knowledge on the mechanisms of algae–microbial interactions. Even for the more well-studied associated bacteria, research has been focused mostly on the effect of specific bacterial species while systematic studies on the effect of microbial communities are still lacking [6,7,11]. Among the main contaminants in microalgal cultures, fungi were also found to be able to influence the production and quality of biomass significantly. Fungi can compromise the algal culture as competitors, predators, or provide a beneficial interaction by becoming symbionts with freshwater and marine microalgae [8,12]. However, the available data for fungi associated with microalgae are very limited. Moreover, it is recognized that the elimination of contaminants, either fungi or bacteria, does not always have the desirable outcome, neither in terms of success nor in terms of cost effectiveness [8]. The co-culture of microalgae with yeasts in the laboratory has shown to be effective in promoting the production of algal biomass and to lead to a higher amount of final relevant bioproducts, as is the case of lipids [13,14,15]. Furthermore, the yeasts capacity to co-produce several interesting compounds as well as to grow on a wide range of substrates makes them very attractive as microbial cell factories envisaging a sustainable circular bioeconomy [15]. The biotechnological exploitation of promising yeast isolates, associated with marine and estuarine environments, is an important objective to be achieved [15].

The marine eustigmatophyte *Microchloropsis gaditana*, formerly known as *Nannochloropsis gaditana*, was renamed in 2015 upon genomic analysis [16]. Nevertheless, the morphology and biochemistry of both genera are very similar. These algae are well appreciated in aquaculture due to their nutritional value, and ability to produce high content of omega-3-long-chain polyunsaturated fatty acids (PUFAs) and pigments. Thus, they are commonly cultivated in marine fish hatcheries as live feed for rotifers and fish larvae [17,18,19]. Species of the genus *Microchloropsis*, including *M. gaditana*, present a high potential to thrive in a broad range of environmental conditions (e.g., variable irradiations), being also considered a promising feedstock for biodiesel production [20,21,22,23]. The oleaginous microalga *M. gaditana*, is considered one of the most promising microalgae species for industrial production, capable of co-producing high-value products [18,19,20,21,22,24]. This is the case of PUFAs, in particular eicosapentaenoic acid (EPA, 20:5ω3), vitamins, and carotenoid pigments with antioxidant activity [18,19,20,21,22,24].

The present study is dedicated to uncovering yeast culturable diversity associated with a large-scale microalgal culture. A scale-up cycle for the industrial cultivation of *M. gaditana*, produced at Necton S. A. facilities (Olhão, Portugal), was followed for three months to isolate and molecularly identify the associated yeast population. A systematic screening for the ability of all the isolates obtained to accumulate lipids and produce biosurfactants was performed during this study under standardized conditions. Results indicate that these isolates are promising for further analysis envisaging yeast biotechnological applications and the study of microalgae–yeasts interactions.

## 2. Materials and Methods

### 2.1. Microchloropsis gaditana Sampling for Yeast Isolation

The marine microalga *Microchloropsis gaditana* was produced on a large scale at Necton S. A. facilities (Olhão, Portugal). The scale-up process started in the laboratory with a 5 L flask (FLF) with aeration (1% CO_2_) and 24 h LED lighting. The FLFs were used to inoculate aerated 80 L columns (CLM) with controlled CO_2_ injection and 24 h LED lighting. CLMs were, in turn, used to inoculate outdoor Flat Panels (FP)—plastic bags of 800 L or 1000 L (with 0.08 m or 0.1 m width) supported by a metal structure with filtered aeration and controlled CO_2_ injection. Finally, several FPs are used to inoculate 19,000 L or 27,000 L Tubular Photobioreactors (PBR).

A scale-up cycle of *M. gaditana* was followed for three months with the collection of 50 mL samples before and after the passage to new reactors. In the PBRs, samples were also collected every week. Before the inoculation of a new reactor, a water sample was collected, whenever possible. In short, the seawater used for microalgae cultivation was collected from Ria Formosa Natural Park (Olhão, Portugal), decanted, ultrafiltered at 0.02 µm, chlorinated, neutralized with thiosulfate and finally treated with UV (except in FLFs, where autoclaved seawater was used). After these treatments, the water was transported from the water deposit, through pipes, to the CLMs, FPs, and PBRs, where the water sampling took place. The water was then supplemented with the culture medium NutriBloom Plus^®^ (Necton, Olhão, Portugal), prior to culture inoculation. A total of 30 *M. gaditana* culture samples and 10 water samples were collected between 23 February 2024 and 20 May 2024. All samples were processed for yeast isolation.

### 2.2. Isolation of M. gaditana-Associated Yeasts

For yeast isolation, the collected samples of microalgal suspensions were serially diluted (10-fold) in 50% (*v*/*v*) Artificial Sea Water [ASW; 23.38 g/L NaCl (PanReac, Barcelona, Spain), 2.41 g/L MgSO_4_·7H_2_O (LabChem, Zelienople, PA, USA), 1.9 g/L MgCl_2_·6H_2_O (Fluka Analytical, Buchs, Switzerland), 1.11 g/L CaCl_2_·2H_2_O (Merck, Darmstadt, Germany), 0.75 g/L KCl (Merck), 0.17 g/L NaHCO_3_ (LabChem) and ddH_2_O]. The original samples and, when adequate, diluted samples were spread (100 µL) onto YPD-Chl-agar [10 g/L yeast extract (VWR Chemicals, Radnor, PA, USA), 20 g/L peptone (BD Gibco, Waltham, MA, USA), 20 g/L glucose (Scharau, Barcelona, Spain), 20 g/L agar (LabChem), and 50% (*v*/*v*) ASW] plates supplemented with 100 µg/mL chloramphenicol (Chl; Sigma, Burlington, MA, USA).

Because of the expected low yeast concentration, 12 mL of the initial microalgal suspensions were centrifuged at 13,000× *g* for 10 min, the pellet resuspended in 400 µL 50% (*v*/*v*) ASW and the concentrated samples were plated onto YPD-Chl-agar plates, as previously described. Additionally, an enrichment culture was prepared using liquid YPD-Chl medium (composition as YPD-Chl-agar medium without agar) with the original sample constituting 10% (*v*/*v*) of the final volume. The culture was incubated at 22 °C with orbital agitation for 48 h. Every 24 h, serial 10-fold dilutions were prepared and plated onto YPD-Chl-agar, as described.

All the plates were incubated at 22 °C for up to 10 days.

Following incubation, the plates were observed, and the colonies were counted. Colonies of different identifiable morphologies, if present, were selected and observed on an Axioplan microscope (1000×) (Zeiss, Oberkochen, Germany) to distinguish yeast colonies from chloramphenicol-resistant bacteria colonies. Isolated colonies (two to four colonies with different morphologies, if possible) were then picked and streaked for purity onto YPD-Chl-agar medium. For long-term storage, isolates (yeasts and Chl-resistant bacteria) are preserved at −80 °C in their isolation medium containing 15% (*v*/*v*) glycerol in the Tecnico_CC (Instituto Superior Técnico Culture Collection).

### 2.3. Molecular Identification of Microbial Isolates

#### 2.3.1. Molecular Identification of Yeast Isolates

For molecular identification of yeast isolates, genomic DNA was extracted with the method described by Lee et al. (2012) [25] and used as a template for amplification by Polymerase Chain Reaction (PCR) of the D1/D2 domain sequence of the 28S ribosomal DNA (rDNA) and the internal transcribed spacer (ITS) region of rDNA. The primer pairs used for D1/D2 and ITS amplification were, respectively, NL-1 (5′-GCATATCAATAAGCGGAGGAAAAG-3′) and NL-4 (5′-GGTCCGTGTTTCAAGACGG-3′), and ITS1 (5′-TCCGTAGGTGAACCTGCGG-3′) and ITS4 (5′-TCCTCCGCTTATTGATATGC-3′), considered effective for the taxonomic identification of yeasts [26]. The PCR protocol included a denaturation step at 98 °C for 30 s, followed by 35 cycles of denaturation at 98 °C for 10 s, annealing at 52 °C for 20 s, and extension at 72 °C for 30 s. A final extension was performed at 72 °C for 10 min. To amplify the DNA fragments, the Phusion™ High-Fidelity DNA Polymerase (Thermofisher Scientific, Waltham, MA, USA) was used. After amplification, the amplicons were purified using the NZYGelpure kit (NZYTech, Lisbon, Portugal) and Sanger sequenced. The yeast species were identified by running a nucleotide BLAST (http://www.ncbi.nlm.nih.gov/blast, assessed on 12 September 2024) with the D1/D2 and ITS-obtained sequences against the core nucleotide NCBI database, and all the D1/D2 and ITS sequences were submitted to GenBank.

#### 2.3.2. Molecular Identification of Chloramphenicol-Resistant Bacterial Isolates

For molecular identification of the 86 Chl-resistant bacterial isolates retained, genomic DNA was extracted by resuspending an isolated colony in 50 µL of nuclease-free water and boiling for 3 min. After a 2 min centrifugation at 9700× *g*, 20 µL of the supernatant was collected, DNA concentration was measured and used as a template for amplification by PCR of the 16S rDNA gene. The primers used were F27 (5′-AGAGTTTGATCGTGGCTCAG-3′) and R1494 (5′-TACGGYTACCTTGTTACGACT-3′), considered effective for the taxonomic identification of bacteria [27]. The PCR protocol included a denaturation step at 95 °C for 5 min, followed by 35 cycles of denaturation at 95 °C for 30 s, annealing at 50 °C for 30 s, and extension at 72 °C for 90 s. A final extension was performed at 72 °C for 10 min. Amplification of the 16S rDNA fragments, purification, sequencing, and isolate identification were performed as described above for the yeast isolates. Sequences were submitted to GenBank.

#### 2.3.3. Phylogenetic Analysis of Yeast Isolates

For the phylogenetic placement of 60 of the 146 yeast isolates obtained in this work, corresponding to one isolate of each species obtained per microalgal culture sample (Supplementary Material Tables S1 and S2, bold), the D1/D2 consensus rDNA sequences were aligned iteratively with the sequences of the type strains for each species, whose GenBank accession numbers are given on Supplementary Material Table S3. Multiple alignment was carried out with Muscle, available in the software MEGA-X v.11. The Mega-X software was also used for the phylogenetic tree construction using the maximum likelihood method of the Kimura 2-parameter model, selected based on the MEGA-X software recommendation considering the data [28]. The confidence level of the clades was estimated using bootstrap analysis with 500 replicates.

### 2.4. Screening the Yeast Isolates for Biosurfactant and Lipid Production

The 60 selected isolates (Supplementary Material Tables S1 and S2, bold) were used for testing biosurfactant and lipid production capacity. Additionally, three *Rhodotorula* strains were used as positive controls for lipid intracellular accumulation. Two of them belong to the species *R. toruloides*, strain IST536, selected in our lab to produce lipids from sugar beet hydrolysates [29], and the multi-stress tolerant mutant IST536 MM15 obtained from IST536 by adaptive laboratory evolution (ALE), which is also a higher lipid producer even in the absence of stress [30,31]. The third strain (IST390) belongs to the species *R. mucilaginosa*, was isolated from sugar beet pulp and was also found to be suitable to produce lipids from pectin-rich biomasses [29].

Yeast cells were pre-cultured in liquid YPD medium (prepared with ddH_2_O) for 24 h with orbital shaking and incubated at 30 °C, except *Vishniacozyma carnescens* isolates, which were incubated at 22 °C due to the susceptibility of the species to higher temperatures [32]. Pre-cultured cells were harvested by centrifugation at 4600× *g* for 5 min at 4 °C and inoculated in 20 mL of minimal medium [6.7 g/L of Yeast Nitrogen Base (BD Difco), 30 g/L of glucose with pH adjusted to 5.5] in 100 mL shake flasks, at an initial optical density at 600 nm (OD600nm) of 1. The cultures were incubated at 30/22 °C with orbital agitation and growth was monitored by measuring OD600nm using a U-2000 HITACHI spectrophotometer (Tokyo, Japan). This medium was filter sterilized using a 0.2 µm filter (Whatman^®^ Puradisc, Maidstone, UK).

#### 2.4.1. Biosurfactant Production Assessment

##### Oil-Displacement Test

Biosurfactant production, after 144 h of cultivation in a minimal medium with glucose as a carbon source, was assessed with the oil displacement test performed as described before [33]. Briefly, 20 mL of water was added to Petri dishes, followed by 1 mL of car oil—(1:46) made of used car oil (to provide color to the mixture) and new car oil (NorAuto ACEA A5/B5 WSS-M2C913-D/5W-30). Finally, 40 μL of cell-free culture supernatant (collected by centrifugation at 3300× *g* for 3 min) was placed onto the oil drop surface. The diameter of oil displacement was measured, with a ruler, after stabilization of the diameter. The test was performed in triplicate and a 1% SDS solution (*w*/*v*) (Sigma) was used as positive control while the cultivation medium was used as negative control.

##### Emulsification Index

The emulsification Index was determined as described in [33] after 144 h of cultivation in minimal medium. Briefly, 800 μL of the culture supernatant was added to 800 μL of olive oil (1:1 ratio) in a 2 mL Eppendorf tube and vortexed for 2 min. After 24 h, the total height (cm) of the liquid and the emulsified layer height (cm) were measured. The emulsification index was calculated by dividing the emulsified layer height by the total height and was expressed as a percentage. The test was performed in triplicate and positive and negative controls were those used for the oil-displacement test.

#### 2.4.2. Assessment of Lipid Accumulation by Nile Red Staining

The assessment of lipid accumulation in yeast cells was carried out based on Nile Red staining [34], routinely used in our lab [29,30]. To reach a final OD600nm of 1.5, the cultures that were prepared for biosurfactant assessment were harvested after 50 h of incubation. Cells were collected by centrifugation (2400× *g*, 3 min) and the pellet resuspended and washed twice with a solution of 0.9% (*w*/*v*) NaCl and the washed pellets resuspended in Phosphate-Buffered Saline [PBS; 1×, pH 7.4, containing 8 g/L NaCl (Sigma), 0.2 g/L KCl (Sigma), 1.44 g/L Na_2_HPO_4_ (Sigma) and 0.24 g/L KH_2_PO_4_ (Sigma)]. These cell suspensions were incubated at 50 °C for 30 min, and a total of 200 µL was transferred to a black 96-well clear bottom plate (Thermo Fisher Scientific), to measure the cell optical density at 600 nm in a POLARstar OPTIMA Microplate Reader (BMG Labtech, Ortenberg, Germany). Cell suspensions were stained with Nile Red (Sigma-Aldrich) at a final concentration of 2.5 µg/mL, and the relative fluorescence units (RFU) were measured using a POLARstar OPTIMA Microplate Reader Software v2.20R2, using the excitation and emission wavelengths of 535 and 625 nm, respectively. To obtain the final values of relative fluorescence units, the background fluorescence values were subtracted: (i) cells with PBS without Nile Red to eliminate buffer autofluorescence, and (ii) PBS with Nile Red. A normalization step of fluorescence to OD600nm was also performed to consider the variation in cell concentration. The three *Rhodotorula* strains mentioned above were used as reference strains and positive controls.

## 3. Results and Discussion

### 3.1. Isolation of Yeasts During M. gaditana Cultivation Scale-Up

To characterize the culturable yeast population, present in 40 samples of *M. gaditana* large-scale cultures produced and collected at Necton S. A. facilities, the microalga scale-up process was followed from the beginning of cultivation, until the end of production. Overall, the culture samples (30) were collected prior to and post-scale-up, and the treated water samples (10), used to fill the new photobioreactors, were collected before inoculation, as described in Section 2.1.

**Figure 1:**
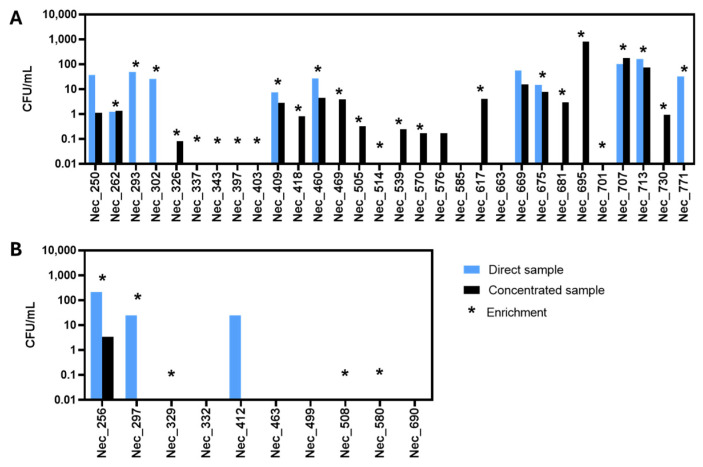
Concentration of culturable yeast cells in the analyzed samples. The concentration, calculated based on colony forming units per milliliter of the original sample (CFU/mL), was obtained for each microalgal culture sample (**A**) and for each water sample (**B**). CFUs/mL were calculated by multiplying/dividing the number of yeast colonies obtained from the original sample (direct sample, blue) or from the concentrated sample (concentrated sample, black), by the dilution/concentration factor, whenever necessary. The stars indicate the samples from which yeast colonies were obtained following an enrichment culture step.

Samples were processed as described in Section 2.2. Yeasts, filamentous fungi and chloramphenicol-resistant (Chl-resistant) bacteria were observed on the inoculated plates. In the original microalgal culture samples (Figure 1A, blue bar) and water samples (Figure 1B, blue bar), yeast abundance was assessed in terms of Colony Forming Units per milliliter (CFU/mL). In the case of the plates inoculated with the enrichment cultures, if yeast colonies were obtained, an asterisk is shown in Figure 1, but no value of CFU/mL is depicted since it does not translate the yeast concentration present in the original sample.

Yeast colonies were detected in 14 of the 40 samples examined by direct inoculation of the original sample in YPD-Chl-agar. In 11 samples, yeast isolation was only possible after the concentration of the original sample and, in 9 samples, yeast colonies were observed only after an enrichment step. No yeast isolates could be obtained when six samples—two microalgae (Nec_585 and Nec_663) and four water (Nec_332, Nec_463, Nec_499 and Nec_690) samples—were analyzed, even after sample concentration or culture enrichment. Surprisingly, in five samples (Nec_293, Nec_302, Nec_771, Nec_297 and Nec_412), no yeast colonies could be obtained on plates inoculated with the concentrated sample even though they were observed and isolated from the corresponding original sample. This discrepancy was possibly due to the higher concentration of filamentous fungi present in the concentrated sample which hampered the isolated growth of yeast colonies. For this reason, those plates were discarded.

During the isolation process, all the plates from the same sample were observed and, whenever possible, 2 to 4 colonies with the same morphology were selected. From the analysis of 30 microalgal culture samples, 125 yeast isolates (Table S1) were retained, while from the 10 water samples, 21 yeast isolates (Table S2) were retained.

### 3.2. Taxonomic Profiling of the Culturable Yeast Population in M. gaditana Cultivation Scale-Up

**Figure 2:**
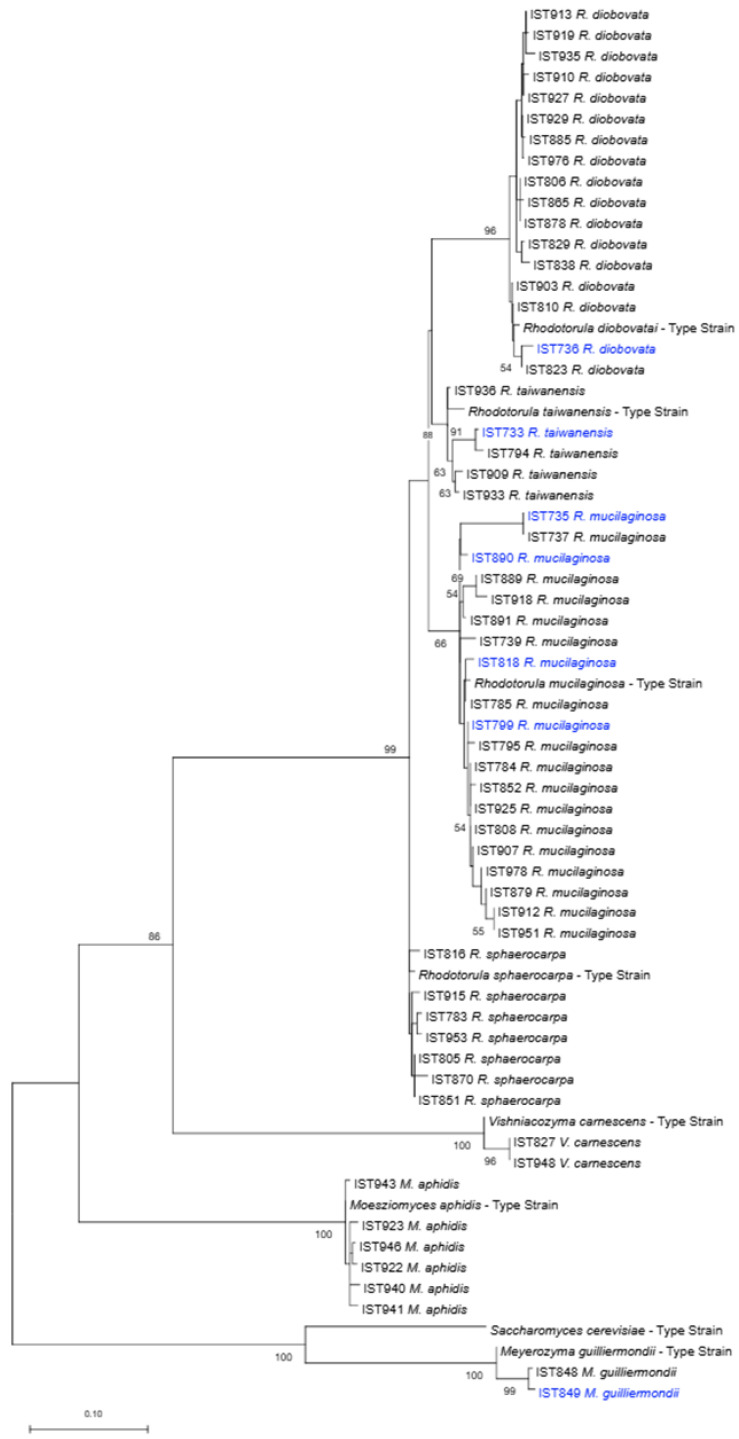
Phylogenetic analysis of yeast isolates obtained in this study (one isolate per species per sample). The taxonomic assignment phylogenetic was based on the alignment of sequences of the D1/D2 domain of the 28S rDNA region, inferred by means of the maximum likelihood method and Kimura 2-parameter model. Sequences from the type strains of the different yeast species were included. Isolates obtained from water samples are indicated in blue. The scale bar indicates the number of expected substitutions per site. The numbers provided at the branches are frequencies in percentage with which a given branch appeared in 500 bootstrap replications.

The 146 yeast isolates obtained were molecularly identified based on the comparison of the D1/D2 and ITS nucleotide sequences with the sequences deposited in the NCBI database and the nucleotide sequences were submitted to GenBank (the accession numbers are displayed in Supplementary Material Tables S1 and S2). Yeast molecular identification at the species level (Tables S1 and S2) is consistent with the phylogenetic analysis performed using D1/D2 consensus region and the maximum likelihood inference method, which placed the isolates close to the type strains for each species (Figure 2).

Most of the 146 yeast isolates identified (99%) belong to six species of the phylum Basidiomycota, including four species of the genus *Rhodotorula*, *R. diobovata* (59 isolates in 17 samples), *R. mucilaginosa* (44 isolates in 21 samples), *R. taiwanensis* (9 isolates in 5 samples), *R. sphaerocarpa* (19 isolates in 7 samples), and two species of two other genera, *Vishniacozyma carnescens* (5 isolates in 2 samples) and *Moesziomyces aphidis* (8 isolates in 6 samples). The species with the highest occurrence were *Rhodotorula mucilaginosa* (30%) and *R. diobovata* (41%). However, the *R. mucilaginosa* isolates were obtained from 21 samples, while *R. diobovata* isolates were obtained from only 17 samples. Such an apparent discrepancy is the result of the similar colony morphology of the *Rhodotorula* species, which may have led to an unbalanced choice of colonies of different species to be retained. Only one yeast species of the phylum Ascomycota was identified, *Meyerozyma guilliermondii* (two isolates in two samples, one of them from a water sample).

Regarding the 10 water samples processed, it was not possible to obtain yeast colonies from four samples. The 21 isolates obtained from the remaining six samples belong to the species *R. mucilaginosa* (11 isolates), *R. diobovata* (5 isolates), *R. taiwanensis* (4 isolates), and *Meyerozyma guilliermondii* (1 isolate). The *R. mucilaginosa* isolates were obtained from five different water samples, while the other species were present in just one sample, each.

Despite the water sterilization processes performed at Necton, including ultrafiltration, chlorination, and UV, in this work it was possible to detect yeasts in some water samples, while they were undetectable when assessed by Necton standard protocols. Considering the sampling conditions (Section 2.1), the microbes might also come from resident contaminants in the cultivation systems. Remarkably, the clustering of the isolates of the same species retrieved from water samples and microalgae culture samples suggests that the water samples, collected in the reactor after treatment and before inoculation, are at the origin of most, if not all, of the cultured yeast species present in microalgae cultures.

The yeast species found in this study have been detected in marine and estuarine environments [35,36,37]. Studies performed in the Portuguese coast and estuarine regions have shown that basidiomycetous yeasts are also prevalent in these environments [38,39]. In particular, red yeasts, characterized by the production of carotenoid pigments, especially those from the genus *Rhodotorula*, are usually found in high proportions, and their appearance cannot be considered a pollution indicator [40]. Some of the most found yeast species in marine and estuarine environments are also *R. mucilaginosa* and *R. diobovata* [36,39]. Other frequently isolated basidiomycetous genera include *Vishniacozyma* (*V. carnescens* was found in our study), *Filobasidium*, *Naganishia*, *Papiliotrema*, *Sterigmatomyces*, and *Cystobasidium* [35,36,37]. Although less common, ascomycetous yeasts are also associated with marine and estuarine environments, especially species belonging to the genera *Candida*, *Meyerozyma* (found in our study), *Kodamaea*, and *Wickerhamomyces* [35,36,37]. These reports are aligned with the results obtained in our study and suggest that in the industrial production of marine microalgal cultures, the yeast population retains some similarity to the one found in wild environments, such as the Ria Formosa, where Necton facilities are located and where the water for the bioreactors is collected from. Interestingly, most of the marine yeasts reported to be isolated from corals and zoanthids also belong to the phylum Basidiomycota, with *R. mucilaginosa* having the highest occurrence [37]. The dominance of certain microbial genera/species associated with specific hosts suggests fundamental ecological relationships.

**Figure 3:**
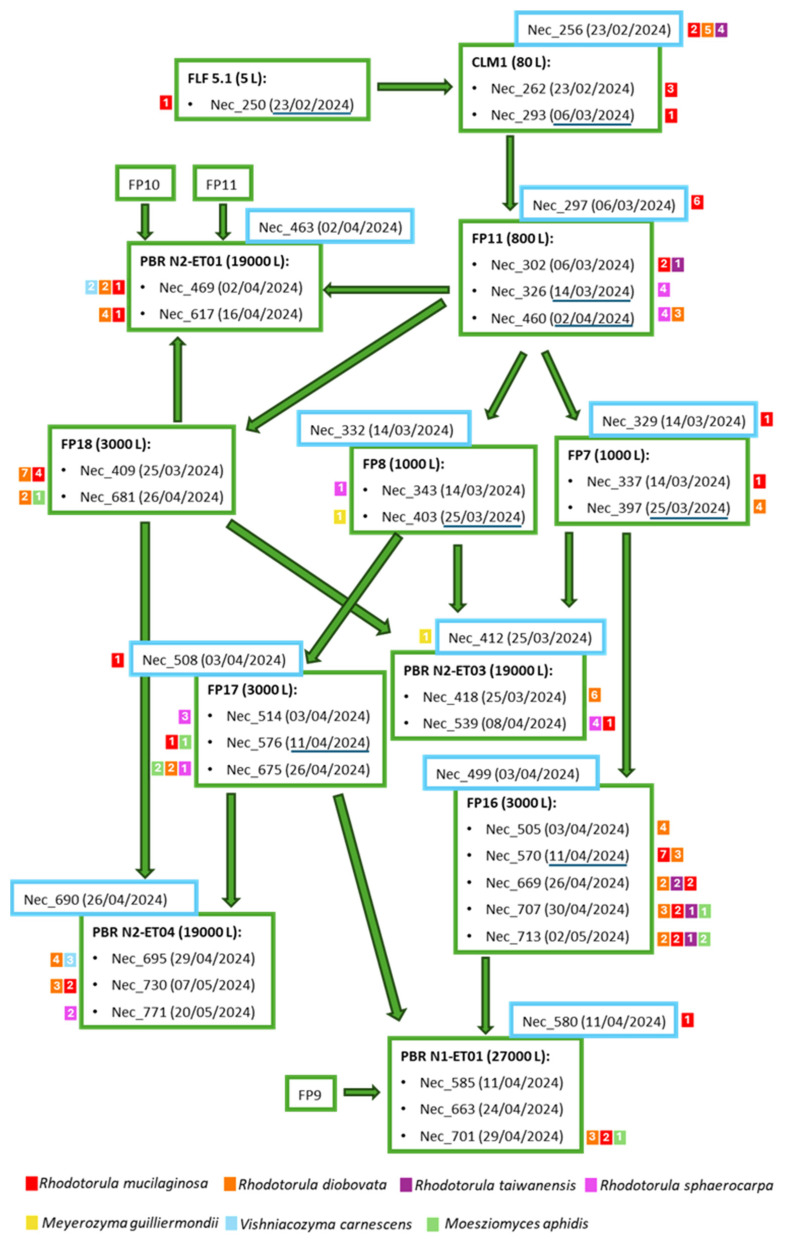
Schematic representation of *Microchloropsis gaditana* production flow. The samples (Nec_) considered in this study are indicated, as well as the collection date within brackets. Water samples are indicated inside blue squares. The reactor from which the samples were collected is in bold. The small colored squares next to each sample indicate the species isolated from the sample (differentiated by color) and the number of isolates retained from each sample (number within the square). The dates of the passages between the reactors are underlined in dark blue. FLF—Five Liter Flasks, CLM—Column, FP—Flat Panel, PBR—Photobioreactor.

To better understand the progression of the isolated yeasts during *M. gaditana* cultivation scale-up, a schematic representation depicting the scale-up process is shown in Figure 3. For the different samples examined, the yeast species and the number of isolates retained for each species are shown. During the scale-up, it was noticed that, based on the established protocol, in general, the number of yeast isolates and yeast species that could be isolated increased. For example, from the 5 L flask sample (Nec_250)—the first step in the scale-up process—only one *R. mucilaginosa* isolate was obtained. However, from subsequent larger industrial photobioreactors, several isolates from the four *Rhodotorula* species were isolated. Unlike the species from the genus *Rhodotorula* that were often present in the different reactor types, the two isolates of *Meyerozyma guilliermondii*, derived from samples collected on the same date, and the isolates from *Vishniacozyma carnescens* were isolated only from the photobioreactors (PBR) and in plates inoculated with the concentrated sample.

An impressively high percentage of red yeasts was obtained during this work (90%, corresponding to all the isolates belonging to the four species of the genus *Rhodotorula*) and raises the question of their significance in biological terms. *Rhodotorula* yeasts can synthesize different carotenoid pigments of high economic value [41]. Carotenoids are pigmented terpenoids, distributed in all taxonomic groups of fungi; they act as antioxidants being able to inactivate oxygen radicals and quench singlet oxygen generated by photosensitized reactions [42]. In particular, *R. mucilaginosa*, one of the most represented yeast species in our study, produces four main pigments: torularhodin, torulene, *β*-carotene, and γ-carotene, which are synthesized, at different rates, depending on the strain [43]. Torularhodin was found to be the pigment with the highest oxidation potential in *Rhodotorula* yeasts being a potent scavenger against singlet oxygen and peroxyl radicals, efficiently inhibiting the peroxidation of lipids [43]. It is recognized that photoprotection by carotenoid pigments is a widespread phenomenon observed in both photosynthetic and non-photosynthetic organisms, from mammalian to fungi [44]. Considering the environmental and ecological conditions of photoautotrophic cultivation of the microalga *M. gaditana* under solar lighting, the abundance of associated red yeasts appears to be likely. Interestingly, a pilot study aiming to evaluate the ability of microalgae and carotenogenic yeast strains to grow and metabolize in co-culture, confirmed that high lipid-producing carotenogenic yeasts (as is the case of *Rhodotorula* strains) and microalgae are capable of symbiotic co-existence with positive impact on biomass growth and lipid yields [45]. These results suggest that the observed presence of the red oleaginous yeasts in association with cultures of the oleaginous microalga *M. gaditana* might have a positive impact on this microalgae growth and nutritional properties, possibly not under natural conditions due to the low abundant yeast population but through controlled contamination. The presence of probiotic yeasts may not only have a promising role in biomass production and nutrition properties of aquatic organisms but also in the immunostimulation of many fish species in modern aquaculture [46].

Chloramphenicol-resistant bacteria were also present in the analyzed samples and were identified based on the 16S rDNA region. More than one-half of the isolates belong to the *Pseudomonas* genus, with preponderance for the species *P. segetis*, *P. marincola* and *P. putida*. The less frequent genera were *Ralstonia*, *Microbacterium*, *Cobetia*, *Pseudoalteromonas*, *Thalassospira*, and *Gordonia*.

### 3.3. Lipid Production by Selected Yeast Isolates

Based on several reports available in the literature, associating the capacity to accumulate intracellular lipids and to produce biosurfactants with strains of the yeast species isolated during this work [29,47,48,49], a rapid screening was performed, envisaging the preliminary assessment of the isolates’ potential, to identify strains deserving further investigation.

**Figure 4:**
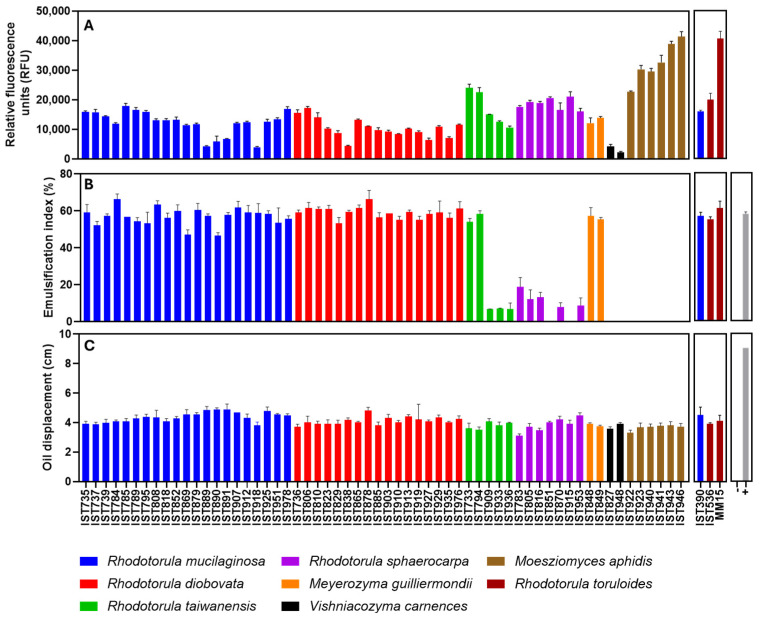
Lipid and biosurfactant production by 60 yeast isolates of the different species retrieved in this study. Lipid production (**A**) was assessed by Nile Red staining and is indicated in relative fluorescence units (RFU). Biosurfactant production was assessed through the emulsification index (**B**) and the oil displacement method (**C**). The chosen isolates were cultivated and the culture tested as described in the Materials and Methods section. For the biosurfactant production assays the positive control (+) was a solution of 1% SDS (*w*/*v*) and the negative control (−) was the growth medium. Three reference strains, *R. toruloides* IST536, *R. toruloides* IST536 MM15 and *R. mucilaginosa* IST390 were also used as reference strains for lipid accumulation. The data shown represent the average of three independent experiments, and the error bars indicate standard deviation.

An isolate of each species per tested sample was selected (Supplementary Material Tables S1 and S2 in bold) for assessment of their capacity to produce lipids. The selected isolates were cultured in shake flasks under standardized conditions in a minimal defined medium with glucose, adjusted to pH 5.5. Cultivation temperature was, in general, 30 °C but *Vishniacozyma carnescens* isolates were cultivated at 22 °C due to the inability of this species to grow at 30 °C. Lipid accumulation by the selected isolates was assessed after 50 h of incubation (Figure 4A) based on previous reports [29,30,47].

Lipid production was assessed based on the Nile Red staining method having, as reference, two promising *Rhodotorula toruloides* strains for lipid production from residual biomasses: the strain IST536 and the evolved multi-stress tolerant strain IST536 MM15 [29,30] and the *R. mucilaginosa* IST390, also suitable for lipid production from pectin-rich residues [29] (Figure 4A). Results indicate that all the isolates of the four *Rhodotorula* species under study exhibit a considerable capacity to accumulate lipids. Interestingly, the production of lipids by the *R. mucilaginosa* isolates obtained in this study was, in general, of the same level as those produced by *R. mucilaginosa* IST390, used as reference [29]. Lipid production by *Meyerozyma guilliermondii* isolates was similar to the isolates from the *Rhodotorula* genus. Poor lipid production values were registered for isolates of *Vishniacozyma carnescens*, a species that, to our best knowledge, was not described as oleaginous. The highest fluorescence values registered were obtained for *Moesziomyces aphidis* isolates under the standardized conditions.

Yeast lipids, and their biotechnological utilization, has recently gathered attention and is a rapidly developing field [47,50], having a broad potential in biotechnological applications in biofuels, oleochemicals, and cosmetics. Some oleaginous yeasts, typically defined as those able to accumulate more than 20% of their cell dry weight as lipids (triacylglycerols), are attracting attention both in research and industry and can be found across several clades [47,50]. Lipid production can be scaled up to satisfy the growing worldwide demand for sustainable alternatives to crude oil to produce liquid biofuels and other chemicals [47,50]. There are also substantial efforts to genetically manipulate yeast metabolism to produce lipids or lipid-derived compounds with high efficiency from low-value substrates, in particular residual biomasses and other wastes [51].

### 3.4. Biosurfactant Production by Selected Yeast Isolates

Biosurfactant production by the previously selected isolates was assessed after 144 h of cultivation (Figure 4B,C) under the growth conditions described for lipid assessment. This standardized incubation time for the screenings was based on previous reports in the literature on the evolution kinetics of these properties in different yeast species [48,52,53]. Biosurfactants are, structurally, a very diverse group of biomolecules (e.g., glycolipids, lipopeptides, lipoproteins) with characteristic physical properties and numerous applications. Because of their amphiphilic properties, biosurfactants reduce surface and interfacial tension and are an eco-friendly alternative to chemical surfactants [49]. Biosurfactants might prevent or delay the formation of biofilms owing to their interfacial and/or antimicrobial properties with activity reported against numerous microorganisms [54]. However, much remains to be revealed about their biological importance and effect on microbial communities in nature.

The biosurfactant production of the selected yeast isolates was assessed with the oil displacement test and the emulsification index. The oil displacement test revealed that all isolates exhibit a substantial activity, with slight differences, while the growth medium (negative control) exhibits no activity and the positive control [a solution of 1% SDS (*w*/*v*)] presented higher values (Figure 4C). However, the emulsification index test allowed the identification of marked differences (Figure 4B) when the same supernatants were tested. The isolates of *R. mucilaginosa*, *R. diobovata,* and *Meyerozyma guilliermondii* formed stable emulsions with analogous and high emulsification indexes (Figure 4B), similar to the positive control [SDS (1% (*w*/*v*)]. The *R. taiwanensis* isolates tested clearly form two groups: two isolates exhibit high emulsification indexes, like the positive control, and three isolates exhibit a poor emulsification capacity. Remarkably, although not so marked, these same two groups were observed for lipid accumulation ability (Figure 4A), and the existence of the two groups is consistent with their phylogenetic relationships (Figure 2). The emulsification capability of the supernatants from *R. sphaerocarpa* was also variable but either poor or non-detectable. No emulsification layer was observed when *Moesziomyces aphidis* isolates were tested.

Based on these results, microalgal cultures, as well as marine and estuarine environments, appear to be promising sampling sites for the isolation of biosurfactant-producing yeasts. The important exception registered in this screening was for all the isolates of the genus *Moesziomyces* (recently renamed from *Pseudozyma*), which includes strains of the marine yeast *Moesziomyces aphidis*, highly efficient producers of mannosylerythritol lipids (MELs) from inexpensive fermentation substrates [52,55]. MELs are among the most promising glycolipids as biosurfactants because of their excellent physicochemical properties, including good emulsification and low critical micelle concentration (CMC) and high environmental compatibility [52,55]. However, under the selected screening conditions, using a defined medium with glucose as a carbon source, this species is able to grow and accumulate intracellular lipids in high amounts, as demonstrated in our study, but is not able to adequately produce MEL, likely due to glucose repression [52]. The high production of intracellular lipids registered for *M. aphidis* isolates under the screening conditions used is also a very promising trait of these isolates. *M. aphidis* lipids are dominated by the fatty acids C16:0, C18:0, C18:1, C18:2, and C18:3 [52], similar to other oleaginous yeast lipids [30,47]. However, the fatty acid precursors of MEL, including C8, C10, and C12, are also detected [52].

Concerning biosurfactant production, *R. mucilaginosa*, *R. diobovata,* and *M. guilliermondii* isolates were found to produce a biosurfactant with high capacity to form stable emulsions. In the literature, the genus *Candida* stands out for its diversity of yeast species producing biosurfactants but, more recently, the genus *Rhodotorula* is also attracting attention with yeasts being considered better producers than bacteria [48]. A screening of more than fifty *Rhodotorula* strains, including *R. mucilaginosa* strains, has shown that *Rhodotorula* species are suitable to produce biosurfactants with antifungal activity [56]. *R. babjevae* produces a heterogeneous sophorolipid, one of the most promising glycolipids biosurfactants, which exhibits excellent oil spreading, emulsifying activities and antifungal activity against plant and human pathogens, which is considered to open up possibilities for the development of efficient and eco-friendly antifungal agents with agricultural and biomedical applications [57]. *M. guilliermondii*’s biosurfactant also has a similar structure to glycolipids [33,58].

Biosurfactants are in demand on the global market, but their biotechnological production is still an emerging field [49]. Currently, the commercial success of biosurfactants is limited by the high cost of production. Therefore, optimized conditions for growth and production, the use of renewable and economically viable substrates, and the availability of microorganisms with higher substrate conversion rates would help the production of more profitable and economically viable biosurfactants [48]. It is essential the discovery of strains producing promising biosurfactants in high yields or new structures leading to strong interfacial activity, high emulsion capacity (valuable for the food industry), and other relevant physicochemical properties in conditions that may turn them commercially viable to replace chemical surfactants.

Besides their biotechnological potential (applications in bioremediation processes, microbial enhanced oil recovery, food industries, cosmetic industries, and biomedical fields), biosurfactants can fulfill various physiological roles and provide different advantages to the producer strains in natural environments. However, much remains to be revealed about the physicochemical effects of biosurfactants produced by a wide range of microorganisms and their biological importance. Biosurfactants possess antimicrobial activity, increase the surface area of water-insoluble substrates by emulsification, increase the bioavailability of hydrophobic nutrients promoting their uptake, and regulate the attachment/detachment of microorganisms to and from surfaces [59]. The antimicrobial activity of biosurfactants can play a role in intraspecific competition, self-defense, and pathogenesis [59], and they can contribute, in many ways, to the efficiency of biocontrol agents. Therefore, research on biosurfactant production conditions and function in a natural environment is essential to the successful development of environmentally friendly biocontrol strategies.

## 4. Conclusions

The diversity of culturable yeasts associated with the industrial production of the microalga *Microchloropsis gaditana* was characterized following the development of a protocol to detect this low-abundance population. The ascomycetous isolates obtained are from the species *Meyerozyma guilliermondii*, whereas the numerous basidiomycetous isolates obtained (99% of all the isolates) belong to four red yeast species of the genus *Rhodotorula* (*R. mucilaginosa*, *R. diobovata*, *R. taiwanensis*, and *R. sphaerocarpa*) and to *Vishniacozyma carnescens* and *Moesziomyces aphidis* species. The yeast populations isolated from microalgal cultures and water samples share yeast species and isolates of the same species that are phylogenetically close, indicating a possible common source. The number of yeast isolates associated with microalgae cultures was found to increase throughout the scale-up process. The capacity of most of the isolates tested to produce biosurfactants and intracellular lipids is promising for biotechnological applications and for the exploitation of yeasts as probiotics in the industrial production of microalgae.

## Data Availability

The original contributions presented in this study are included in the article and Supplementary Material. Further inquiries can be directed to the corresponding author.

## Supplementary Materials

The following supporting information can be downloaded at: https://www.mdpi.com/article/10.3390/jof11030228/s1, Table S1: Yeast isolates retained for further studies following isolation from the different samples of *Microchloropsis gaditana* cultures schematically represented in Figure 3. Information on the isolate ID, isolation conditions, species, GenBank NCBI accession number of the D1/D2 and ITS consensus sequences deposited, is provided. The isolates represented in bold are those selected for biosurfactants and lipid production screening; Table S2: Yeast isolates retained for further studies following isolation from the different water samples used for *Microchloropsis gaditana* cultivation. Information on the isolate ID, isolation conditions, species, GenBank NCBI accession number of the D1/D2 and ITS consensus sequences deposited, is provided. The isolates represented in bold are those selected for biosurfactants and lipid production screening; Table S3: GenBank NCBI accession number of the D1/D2 of the type strains of the different species isolated.

## References

[1] Dolganyuk V., Belova D., Babich O., Prosekov A., Ivanova S., Katserov D., Patyukov N., Sukhikh S. (2020). Microalgae: A Promising Source of Valuable Bioproducts. Biomolecules.

[2] Su M., Bastiaens L., Verspreet J., Hayes M. (2023). Applications of Microalgae in Foods, Pharma and Feeds and Their Use as Fertilizers and Biostimulants: Legislation and Regulatory Aspects for Consideration. Foods.

[3] Osorio-Reyes J.G., Valenzuela-Amaro H.M., Pizaña-Aranda J.J.P., Ramírez-Gamboa D., Meléndez-Sánchez E.R., López-Arellanes M.E., Castañeda-Antonio M.D., Coronado-Apodaca K.G., Gomes Araújo R., Sosa-Hernández J.E. (2023). Microalgae-Based Biotechnology as Alternative Biofertilizers for Soil Enhancement and Carbon Footprint Reduction: Advantages and Implications. Mar. Drugs.

[4] Kofi Tulashie I.S., Iddrisu M., Miyittah M., Atiiga A.-W., Mensah S., Dadzie A. (2023). Large scale production of lipid for biodiesel from green microalgae using wastewater. Chem. Eng. Commun..

[5] Narala R.R., Garg S., Sharma K.K., Thomas-Hall S.R., Deme M., Li Y., Schenk P.M. (2016). Comparison of Microalgae Cultivation in Photobioreactor, Open Raceway Pond, and a Two-Stage Hybrid System. Front. Energy Res..

[6] Hosseini H., Saadaoui I., Cherif M., Amir Siddiqui S., Sayadi S. (2024). Exploring the dynamics of algae-associated microbiome during the scale-up process of *Tetraselmis* sp.. microalgae: A metagenomics approach. Bioresour. Technol..

[7] Lian J., Wijffels R.H., Smidt H., Sipkema D. (2018). The effect of the algal microbiome on industrial production of microalgae. Microb. Biotechnol..

[8] Laezza C., Salbitani G., Carfagna S. (2022). Fungal Contamination in Microalgal Cultivation: Biological and Biotechnological Aspects of Fungi-Microalgae Interaction. J. Fungi.

[9] Samoraj M., Çalış D., Trzaska K., Mironiuk M., Chojnacka K. (2024). Advancements in algal biorefineries for sustainable agriculture: Biofuels, high-value products, and environmental solutions. Biocatal. Agric. Biotechnol..

[10] Gururani P., Bhatnagar P., Kumar V., Vlaskin M.S., Grigorenko A.V. (2022). Algal Consortiums: A Novel and Integrated Approach for Wastewater Treatment. Water.

[11] Steinrücken P., Jackson S., Müller O., Puntervoll P., Kleinegris D.M.M. (2023). A closer look into the microbiome of microalgal cultures. Front. Microbiol..

[12] Salvatore M.M., Carraturo F., Salbitani G., Rosati L., De Risi A., Andolfi A., Salvatore F., Guida M., Carfagna S. (2023). Biological and metabolic effects of the association between the microalga *Galdieria sulphuraria* and the fungus *Penicillium citrinum*. Sci. Rep..

[13] Arora N., Patel A., Mehtani J., Pruthi P.A., Pruthi V., Poluri K.M. (2019). Co-culturing of oleaginous microalgae and yeast: Paradigm shift towards enhanced lipid productivity. Environ. Sci. Pollut. Res. Int..

[14] Xue F., Miao J., Zhang X., Tan T. (2010). A new strategy for lipid production by mix cultivation of *Spirulina platensis* and *Rhodotorula glutinis*. Appl. Biochem. Biotechnol..

[15] Mapelli-Brahm P., Gómez-Villegas P., Gonda M.L., León-Vaz A., León R., Mildenberger J., Rebours C., Saravia V., Vero S., Vila E. (2023). Microalgae, Seaweeds and Aquatic Bacteria, Archaea, and Yeasts: Sources of Carotenoids with Potential Antioxidant and Anti-Inflammatory Health-Promoting Actions in the Sustainability Era. Mar. Drugs.

[16] Fawley M.W., Jameson I., Fawley K.P. (2015). The phylogeny of the genus *Nannochloropsis* (Monodopsidaceae, Eustigmatophyceae), with descriptions of *N. australis* sp. nov. and *Microchloropsis* gen. nov. Phycologia.

[17] Rocha J.M.S., Garcia J.E.C., Henriques M.H.F. (2003). Growth aspects of the marine microalga *Nannochloropsis gaditana*. Biomol. Eng..

[18] Cecchin M., Berteotti S., Paltrinieri S., Vigliante I., Iadarola B., Giovannone B., Maffei M.E., Delledonne M., Ballottari M. (2020). Improved lipid productivity in *Nannochloropsis gaditana* in nitrogen-replete conditions by selection of pale green mutants. Biotechnol. Biofuels.

[19] Martínez R., García-Beltrán A., Kapravelou G., Mesas C., Cabeza L., Perazzoli G., Guarnizo P., Rodríguez-López A., Andrés Vallejo R., Galisteo M. (2022). In Vivo Nutritional Assessment of the Microalga *Nannochloropsis gaditana* and Evaluation of the Antioxidant and Antiproliferative Capacity of Its Functional Extracts. Mar. Drugs.

[20] Scholz M.J., Weiss T.L., Jinkerson R.E., Jing J., Roth R., Goodenough U., Posewitz M.C., Gerken H.G. (2014). Ultrastructure and composition of the *Nannochloropsis gaditana* cell wall. Eukaryot. Cell.

[21] Ye Y., Liu M., Yu L., Sun H., Liu J. (2024). *Nannochloropsis* as an Emerging Algal Chassis for Light-Driven Synthesis of Lipids and High-Value Products. Mar. Drugs.

[22] San Pedro A., González-López C.V., Acién F.G., Molina-Grima E. (2015). Outdoor pilot production of *Nannochloropsis gaditana*: Influence of culture parameters and lipid production rates in raceway ponds. Algal Res..

[23] Ferrer-Ledo N., Stegemüller L., Janssen M., Wijffels R.H., Barbosa M.J. (2023). Growth and fatty acid distribution over lipid classes in *Nannochloropsis oceanica* acclimated to different temperatures. Front. Plant Sci..

[24] Henriques M., Rocha J.M.S. (2009). Influence of light: Dark Cycle in the Cellular Composition of Nannochloropsis Gaditana.

[25] Lee C.K., Araki N., Sowersby D.S., Lewis L.K. (2012). Factors affecting chemical-based purification of DNA from *Saccharomyces cerevisiae*. Yeast.

[26] Kurtzman C.P., Robnett C.J. (1998). Identification and phylogeny of ascomycetous yeasts from analysis of nuclear large subunit (26S) ribosomal DNA partial sequences. Antonie Van Leeuwenhoek.

[27] Weisburg W.G., Barns S.M., Pelletier D.A., Lane D.J. (1991). 16S ribosomal DNA amplification for phylogenetic study. J. Bacteriol..

[28] Tamura K., Stecher G., Kumar S. (2021). MEGA11: Molecular Evolutionary Genetics Analysis Version 11. Mol. Biol. Evol..

[29] Martins L.C., Palma M., Angelov A., Nevoigt E., Liebl W., Sá-Correia I. (2021). Complete Utilization of the Major Carbon Sources Present in Sugar Beet Pulp Hydrolysates by the Oleaginous Red Yeasts *Rhodotorula toruloides* and *R. mucilaginosa*. J. Fungi.

[30] Fernandes M.A., Mota M.N., Faria N.T., Sá-Correia I. (2023). An Evolved Strain of the Oleaginous Yeast *Rhodotorula toruloides*, Multi-Tolerant to the Major Inhibitors Present in Lignocellulosic Hydrolysates, Exhibits an Altered Cell Envelope. J. Fungi.

[31] Antunes M., Mota M.N., Sá-Correia I. (2024). Cell envelope and stress-responsive pathways underlie an evolved oleaginous *Rhodotorula toruloides* strain multi-stress tolerance. Biotechnol Biofuels Bioprod.

[32] Buzzini P., Turchetti B., Yurkov A. (2018). Extremophilic yeasts: The toughest yeasts around?. Yeast.

[33] Sharma P., Sangwan S., Kaur H. (2019). Process parameters for biosurfactant production using yeast *Meyerozyma guilliermondii* YK32. Environ. Monit. Assess..

[34] Morales-Palomo S., González-Fernández C., Tomás-Pejó E. (2022). Prevailing acid determines the efficiency of oleaginous fermentation from volatile fatty acids. J. Environ. Chem. Eng..

[35] Monapathi M.E., Bezuidenhout C.C., James Rhode O.H. (2020). Aquatic yeasts: Diversity, characteristics and potential health implications. J. Water Health.

[36] Fell J.W. (2012). Yeasts in marine environments. Marine Fungi.

[37] Kaewkrajay C., Chanmethakul T., Limtong S. (2020). Assessment of Diversity of Culturable Marine Yeasts Associated with Corals and Zoanthids in the Gulf of Thailand, South China Sea. Microorganisms.

[38] de Almeida J.M.G.C.F. (2005). Yeast community survey in the Tagus estuary. FEMS Microbiol. Ecol..

[39] Gadanho M., Almeida J.M., Sampaio J.P. (2003). Assessment of yeast diversity in a marine environment in the south of Portugal by microsatellite-primed PCR. Antonie Van Leeuwenhoek.

[40] Hagler A.N., Mendonça-Hagler L.C. (1981). Yeasts from marine and estuarine waters with different levels of pollution in the state of rio de janeiro, Brazil. Appl Environ. Microbiol.

[41] Moliné M., Libkind D., van Broock M., Barredo J.-L. (2012). Production of Torularhodin, Torulene, and β-Carotene by *Rhodotorula* Yeasts. Microbial Carotenoids from Fungi: Methods and Protocols.

[42] Sandmann G. (2022). Carotenoids and Their Biosynthesis in Fungi. Molecules.

[43] Moliné M., Flores M.R., Libkind D., del Carmen Diéguez M., Farías M.E., van Broock M. (2010). Photoprotection by carotenoid pigments in the yeast *Rhodotorula mucilaginosa*: The role of torularhodin. Photochem. Photobiol. Sci..

[44] Will O.A., Scovel C.A., Krinsky N.I., Mathews-Roth M.M., Taylor R.F. (1989). Photoprotective Functions of Carotenoids. Carotenoids: Chemistry and Biology.

[45] Szotkowski M., Holub J., Šimanský S., Hubačová K., Sikorová P., Mariničová V., Němcová A., Márová I. (2021). Bioreactor Co-Cultivation of High Lipid and Carotenoid Producing Yeast *Rhodotorula kratochvilovae* and Several Microalgae under Stress. Microorganisms.

[46] Jamal M., Mahdy M., Harbi M., Al-Mur B., Haque M. (2022). Use of yeasts in aquaculture nutrition and immunostimulation: A review. J. Appl. Biol. Biotechnol..

[47] Mota M.N., Múgica P., Sá-Correia I. (2022). Exploring Yeast Diversity to Produce Lipid-Based Biofuels from Agro-Forestry and Industrial Organic Residues. J. Fungi.

[48] Fernandes N.d.A.T., Simões L.A., Dias D.R. (2023). Biosurfactants Produced by Yeasts: Fermentation, Screening, Recovery, Purification, Characterization, and Applications. Fermentation.

[49] Puyol McKenna P., Naughton P.J., Dooley J.S.G., Ternan N.G., Lemoine P., Banat I.M. (2024). Microbial Biosurfactants: Antimicrobial Activity and Potential Biomedical and Therapeutic Exploits. Pharmaceuticals.

[50] Passoth V., Sibirny A.A. (2017). Lipids of Yeasts and Filamentous Fungi and Their Importance for Biotechnology. Biotechnology of Yeasts and Filamentous Fungi.

[51] Jiang W., Li C., Li Y., Peng H. (2022). Metabolic Engineering Strategies for Improved Lipid Production and Cellular Physiological Responses in Yeast *Saccharomyces cerevisiae*. J. Fungi.

[52] Yu G., Wang X., Zhang C., Chi Z., Chi Z., Liu G. (2022). Efficient production of mannosylerythritol lipids by a marine yeast *Moesziomyces aphidis* XM01 and their application as self-assembly nanomicelles. Mar. Life Sci. Technol..

[53] Morita T., Fukuoka T., Imura T., Kitamoto D. (2009). Production of glycolipid biosurfactants by basidiomycetous yeasts. Biotechnol. Appl. Biochem..

[54] Jimoh A.A., Booysen E., van Zyl L., Trindade M. (2023). Do biosurfactants as anti-biofilm agents have a future in industrial water systems?. Front. Bioeng. Biotechnol..

[55] Niu Y., Wu J., Wang W., Chen Q. (2019). Production and characterization of a new glycolipid, mannosylerythritol lipid, from waste cooking oil biotransformation by *Pseudozyma aphidis* ZJUDM34. Food Sci. Nutr..

[56] Gharaghani M., Zarei Mahmoudabadi A., Halvaeezadeh M. (2019). Evaluation of Laboratory-Produced Biosurfactant by *Rhodotorula* Species and Its Antifungal Activity. Jundishapur J. Nat. Pharm. Prod..

[57] Sen S., Borah S.N., Bora A., Deka S. (2017). Production, characterization, and antifungal activity of a biosurfactant produced by *Rhodotorula babjevae* YS3. Microb. Cell Fact..

[58] Camargo F.P., Menezes A.J.d., Tonello P.S., Dos Santos A.C.A., Duarte I.C.S. (2018). Characterization of biosurfactant from yeast using residual soybean oil under acidic conditions and their use in metal removal processes. FEMS Microbiol. Lett..

[59] Ron E.Z., Rosenberg E. (2001). Natural roles of biosurfactants. Environ. Microbiol..

